# Model selection in the reconstruction of regulatory networks from time-series data

**DOI:** 10.1186/1756-0500-2-68

**Published:** 2009-05-05

**Authors:** Eugene Novikov, Emmanuel Barillot

**Affiliations:** 1 Service Bioinformatique, Institut Curie, 26 Rue d'Ulm, 75248 Paris Cedex 05, France

## Abstract

**Background:**

A widely used approach to reconstruct regulatory networks from time-series data is based on the first-order, linear ordinary differential equations. This approach is justified if it is applied to system relaxations after weak perturbations. However, weak perturbations may not be informative enough to reveal network structures. Other approaches are based on specific models of gene regulation and therefore are of limited applicability.

**Findings:**

We have developed a generalized approach for the reconstruction of regulatory networks from time-series data. This approach uses elements of control theory and the state-space formalism to approximate interactions between two observable nodes (*e.g*. measured genes). This leads to a reconstruction model formulated in terms of integral equations with flexible kernel functions. We propose a library of kernel functions that can be used for the first insights into network structures.

**Conclusion:**

We have found that the appropriate kernel function significantly increases the accuracy of network reconstruction. The best kernel can be selected using prior information on a few nodes' interactions. We have shown that it may be already possible to select models ensuring reasonable performance even with as small as two known interactions. The developed approaches have been tested with simulated and experimental data.

## Findings

Two sources of experimental data are generally used in the reconstruction of regulatory networks: steady-state and time-series experiments. Steady-state data [1,2] are generated by measuring the expression levels of every gene (or protein concentrations) when a system relaxes into a steady state after a perturbation. There are many publications [3-5] reporting different methods for the network reconstruction from the steady-state data. Time-series data represent the expression levels measured at a number of time points following global or local perturbations of a system [6,7]. If these perturbations do not bring the system far from a steady state, the relaxation into the steady state is approximated by a set of the first-order, linear ordinary differential equations (LODE) [6,8,9]. Time-series experiments do not require as many perturbations as steady-state experiments, thus avoiding perturbations that may be not easy to design [10,11]. Moreover, analysis of time-series data allows us to investigate the dynamics of regulatory interactions, which is not possible from the steady-state data.

However, it has been shown [4,5] that the network reconstruction is more difficult from the time-series data than from the steady-state data. The authors have envisaged two possibilities to improve the reconstruction. One is to collect more time series from additional perturbations. The other one is to perform time-series experiments where an investigated system demonstrates richer dynamics. The latter case is advantageous because it may generate more informative data without performing extra experiments. This can be done either by applying stronger perturbations or by monitoring system dynamics controlled by internal factors (*e.g*. cell-cycle processes). In both cases, the LODE models can hardly be justified as it is difficult to ensure that a system does not strongly deviate from a steady state. More sophisticated system dynamics needs more detailed formalizations on gene/molecular interactions. Many attempts to improve the basic LODE model can be found in recent publications [12-14]. In most cases, the authors suggest to model the combined regulatory effect of a number of regulatory factors by a particular non-linear function. Additionally, the second-order differential equations are sometimes invoked to reproduce gene expression profiles [14,15].

In this paper, we are looking for a generic approach to approximate interactions between the observable nodes in a network. The generic approach allows us to systematically apply specific models and, eventually, to define the most appropriate model using available experimental data and, possibly, prior knowledge on the nodes' interactions. The developed approaches were tested with simulated and experimental data.

### Mathematical framework

**Figure 1:**
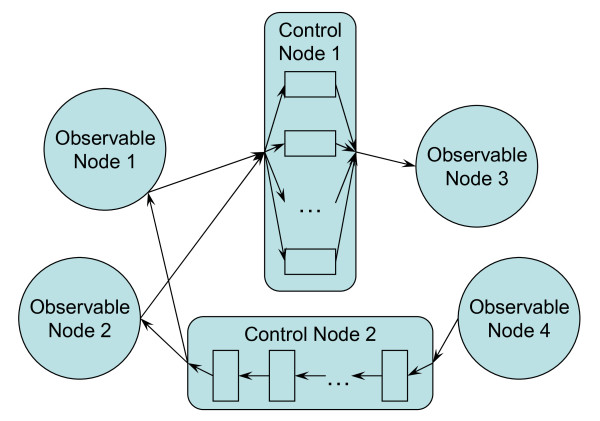
**Regulatory network with four observable and two control nodes**.

We apply elements of control theory [16] to develop a generalized model of the network dynamics. A regulatory network (Fig. 1) is represented as a bipartite graph with two types of nodes: observable nodes reproducing measurable characteristics (*e.g*. gene expression levels), and non-observable, or control, nodes controlling the interactions between the observable nodes. Each control node *i *can be modelled as:

(1)

where *F*_*i *_is a functional reproducing behaviour, *Y*_*I*_(·), of a set of observable nodes *I *based on signals, *Y*_*O*_(·), from a, possibly different, set of observable nodes *O*, and *W*_*i *_is a vector of "internal" parameters of control node *i*. Note that some non-trivial behaviour can be assigned to the observable nodes as well. It may account for instrumental distortions, specifics of image processing, normalization, etc.

The goal of the network reconstruction is to identify parameters *W*_*i *_encoding for the interactions between the observable nodes. For that, functional *F*_*i *_in (1) has to be further developed. It is frequently assumed that the cooperative regulatory contribution from different observable nodes is a sum of the contributions from each node, so that equation (1) can be written as:

(2)

where *n *is the number of observable nodes, *y*_*i*_(*t*) is the measured response of observable node *i*, *F*_*ij *_is a functional characterized by a set of parameters *W*_*ij *_converting measured profile, *y*_*j*_(*t*), at node *j *to measured profile, *y*_*i*_(*t*), at node *i*, and *b*_*i*_(*t, t*_*0*_) is the output of non-regulated observable node *i*. We consider pair-wise controls *F*_*ij *_as linear, continuous, time-invariant, finite-dimensional, single input-single output control systems that can be modelled using the state-space formalism:

(3)

where *X*_*ij*_(*t*) is the state vector and *A*_*ij *_is the state matrix, *y*_*j*_(*t*) is the input value and *B*_*ij *_is the input vector, *y*_*j*_(*t*) is the output value and *C*_*ij *_is the output vector. We also assume that *F*_*ij *_are in a steady state prior to the input perturbation *y*_*j*_(*t*) starting at time *t *= *t*_*0*_, that is *X*_*ij*_(*t*_*0*_) = 0. Integrating (3) and combining *n *regulatory inputs as in (2) yields

(4)

with *w*_*ij*_(*t*) = *C*_*ij*_*exp*(*tA*_*ij*_)*B*_*ij *_representing the influence of node *j *on the regulation of node *i*. Although every link (control node) is unique and should be modelled in a specific way, little prior knowledge on molecular interactions does not allow us to postulate specific models for every link. Therefore, we are looking for universal models that can approximate any control node.

The LODE regulatory model is widely used in the network reconstruction [6,8,9]. It can be obtained from (4), if we set *w*_*ij*_(*t*) = *const *= *w*_*ij *_and *b*_*i*_(*t, t*_*0*_) = *const*×*t *= *b*_*i*_*t*:

(5)

This model approximates system relaxation into a steady state after a small perturbation. However, it is difficult to confirm that perturbations are small enough to justify model (5).

Equation (4) allows us to create a number of less restrictive models that can cover broader spectrum of dynamical behaviours. These models can integrate prior knowledge or can be further refined in experimental data analysis. In this report, we use the following representations for *w*_*ij*_(*t*):

(6)

(7)

(8)

where *L *is the number of terms, *u*_*l*, *ij *_are the coefficients encoding for the regulation of node *i *by node *j *and *τ*_*l *_are the characteristics times that can be either set as prior values or estimated from experimental data. The background functions *b*_*i*_(*t, t*_*0*_) can also be developed, but we will keep them constant as, with little data, more complicated models for *b*_*i*_(*t, t*_*0*_) can fit the data without identifying any link.

We have devised a library of eight models (Table 1) to be tested and compared. Rationale for using the selected kernel functions is given in [Additional file 1].

**Table 1:** Kernel functions

Equation	*w_*ij*_(t)*	Model
(6)	*u*_1, *ij*_	P1
	
	*u*_1, *ij *_+ *u*_2, *ijt*_	P2

(7)	*u*_1, *ij*_exp{-*t*/(0.1*T*)}	E1
	
	*u*_1, *ij*_exp{-*t*/(0.9*T*)}	E2
	
	*u*_1, *ij*_exp{-*t*/(0.1*T*)} + *u*_2, *ij*_exp{-*t*/(0.9*T*)}	E3

(8)	*u*_1, *ij *_(1 + *t*/(0.1*T*))^-1^	I1
	
	*u*_1, *ij *_(1 + *t*/(0.9*T*))^-1^	I2
	
	*u*_1, *ij*_(1 + *t*/(0.1*T*))^-1 ^+ *u*_2, *ij*_(1 + *t*/(0.9*T*))^-1^	I3

Discussion on the parameter identifiability for the developed models can be found in [Additional file 2].

Network reconstruction is done by fitting the developed models to experimental data. Among different fitting strategies [17], the forward selection (FS) algorithm has shown reasonable performance, in particular for sparse networks, and therefore, it has been adopted in this paper. We refer to [18] for the details on the implementation of the FS algorithm. A more robust modification of the FS algorithm has also been tested as described in [Additional file 3].

We can use prior knowledge on the nodes' interactions to select the best network reconstruction model from the pre-defined library (Table 1). We look for the kernel function *w*_*ij*_(*t*) that reconstructs the prior links with the highest accuracy. The description of the adaptive model selection (AMS) algorithm can be found in [Additional file 4].

### Testing

We compared the performances of the eight kernel functions from Table 1 as well as the LODE regulatory model (5) using simulated and experimental data. Three artificial systems were used for testing: the oscillating network in *E. coli*, called repressilator [19], the mitogen-activated protein kinase (MAPK) cascade [20] and the glycolysis pathway in yeast [21]. We also used the yeast (*Saccharomyces cerevisiae*) cell cycle microarray time-series data [22] to demonstrate applicability of the developed approach to real experimental data. The positive predictive value (PPV) and sensitivity (Se) were applied to estimate the performance. Further details on the artificial and real systems used for testing and description of the testing procedure can be found in [Additional file 5].

**Figure 2:**
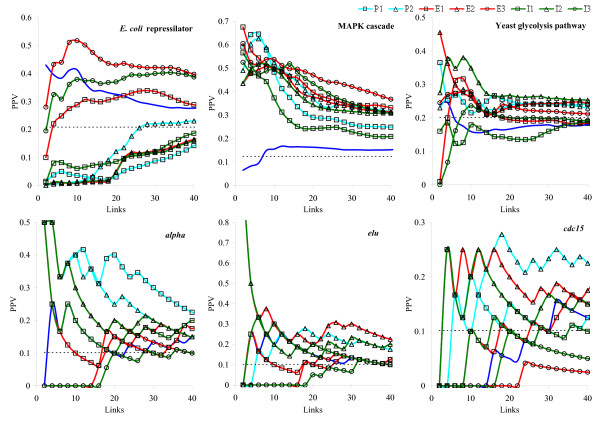
**The average dependencies of PPV on the total number of links for the three artificial systems and for the three yeast cell cycle microarray time-series datasets**. Blue line corresponds to the LODE model and dashed black line corresponds to random prediction. Confidence intervals for the obtained estimates are too narrow to be recognizable in the graphs and therefore not shown.

The dependencies of PPV on the total number of links are presented in Fig. 2. The Se values at 50 generated links are collected in Table 2. Among the three artificial systems, the choice of a model was the most critical for the *E. coli *repressilator. In this case, the best reconstruction was achieved with the bi-exponential E3 model. The LODE model performed better than random reconstruction but still worse than E3. All tested kernels were significantly better than random link assignment for the MAPK cascade. All kernels also outperformed the LODE model in this case. However, there is still a notable (and statistically significant) difference between the kernels. The yeast glycolysis network (Fig. 2c) was the most difficult to reconstruct because many times series were similar and hardly distinguishable by the reconstruction algorithm. Nevertheless, several models (P1, P2, E2, E3, and I2) demonstrated the performance different from random. The LODE model could not outperform the random prediction in this case.

**Table 2:** Se at 50 generated links for the three artificial systems (*E. COLI *repressilator (A), MAPK cascade (B) and yeast glycolysis pathway (C)) and three yeast cell cycle microarray time-series datasets

Models	*A*	B	C	*alpha*	*elu*	*cdc15*
LODE	0.46	0.12	0.16	0.23	0.19	0.27
P1	0.32	0.19	0.20	0.35	0.42	0.27
P2	0.41	0.23	0.18	0.35	0.31	0.35
E1	0.47	0.25	0.16	0.38	0.31	0.23
E2	0.32	0.24	0.20	0.31	0.35	0.31
E3	0.60	0.27	0.17	0.15	0.27	0.08
I1	0.35	0.18	0.18	0.31	0.23	0.15
I2	0.32	0.24	0.21	0.27	0.35	0.27
I3	0.59	0.23	0.16	0.19	0.19	0.12

For the artificial systems, the Se values were averaged over 100 runs of the simulation procedure. Model definitions (P1, P2, E1, E2, E3, I1, I2 and I3) are given in Table 1.

For the yeast cell cycle time-series data, the polynomial models (P1 and P2) were the most powerful. For the *alpha *dataset and for the *elu *dataset, P1 had the highest performance whereas P2 was the most accurate for *cdc15*. Note that, in each case, the best performing models (P1 and P2) also outperformed the LODE model. Comparing different experiments, we see that *cdc15 *led to less accurate predictions. This indicates that this experiment requires more elaborated reconstruction models or more representative datasets.

From Fig. 2 and Table 2, we can conclude that the "optimal" models were different for the artificial and real systems. The obtained results suggest that no unique model exists to ensure reasonable performance for different systems and therefore the most appropriate models should be searched for each system.

**Figure 3:**
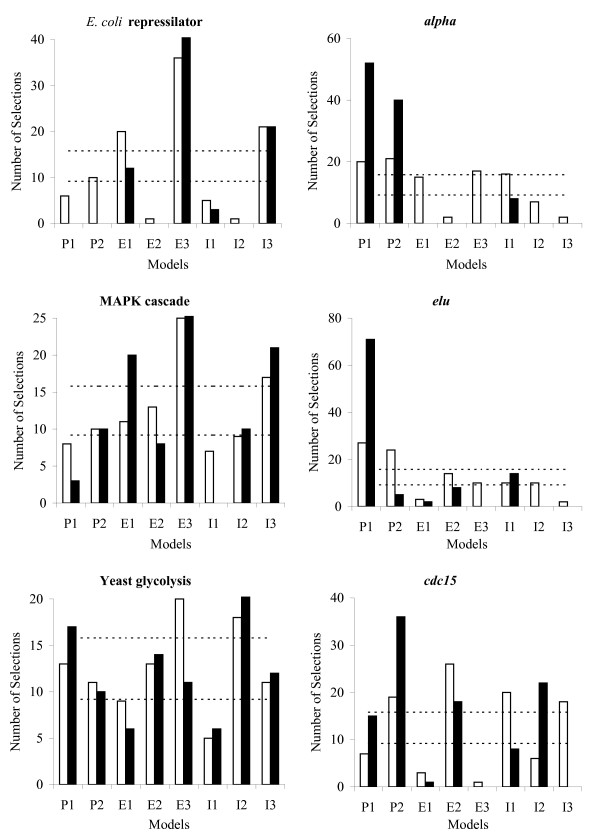
**Adaptive model selection**. Number of times each model from Table 1 has been selected in 100 runs of the simulation procedure by the AMS algorithm based on 2 (empty bars) and 10 (filled bars) prior links. Confidence intervals for the random model selection are indicated by dashed lines.

We applied the AMS algorithm [Additional file 4] to the same three artificial systems and three experimental datasets. As at each run the prior links were different, the selected model might also be different. Therefore, we counted number of times each model from Table 1 was selected in the 100 runs. The results for 2 and 10 prior links are shown in Fig. 3. We found that the higher performing models from Fig. 2 were selected more often than the lower performing ones. Moreover, reasonable model recognition could be already achieved with only two prior links. As expected, the increase in the number of prior links led to better model identification.

However, in some cases with two prior links, the AMS algorithm relatively often selected the models that were rather poor as judged by the results presented in Fig. 2. For example, for the artificial yeast glycolysis pathway or real *alpha *dataset, the bi-exponential E3 model was selected almost as often as other, better performing, models. This indicates that the E3 model was more adequate just for certain links and not for any link in the networks. Therefore, we can conclude that the network reconstruction model should be link-specific, that is different models may be assigned to different links.

**Figure 4:**
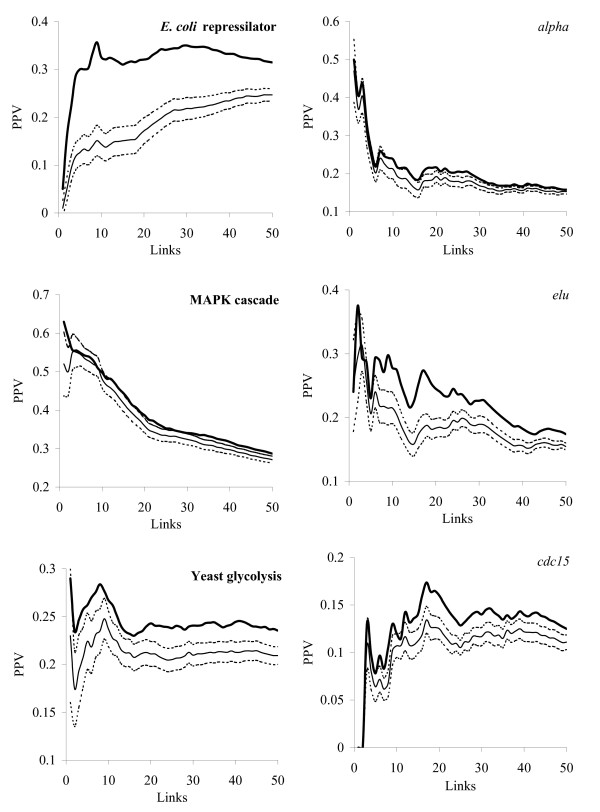
**The dependencies of PPV on the total number of links for the AMS algorithm (with two prior links)**. Thick line – PPV by the AMS algorithm; thin line – PPV after random model selection. Confidence intervals for PPV after random model selection are shown as dashed lines.

As the AMS algorithm may select poor performing models, the overall performance of the network reconstruction is lower than for the best performing model. However, even with as small as two prior links, AMS is already better than random model selection, as illustrated in Fig. 4. If the performance of different models is not very different (as for the MAPK cascade), the prediction of the AMS algorithm is close to random. If, however, a certain model demonstrates clear advantage (as, for example, for the *E. coli *repressilator), the AMS algorithm can identify this model leading to the performance substantially higher than by random selection.

The performance of the AMS algorithm using independent set of artificial data described in [5] is presented in [Additional file 6].

## Conclusion

We have presented a generalized approach for the regulatory network reconstruction, that gives us an easy possibility to create and to test different inference models and, potentially, to identify appropriate models from experimental data. We have shown that even with as small as two prior links it is already possible to select models ensuring reasonable performance. Further discussion and perspectives for further research are given in [Additional file 7].

## Competing interests

The authors declare that they have no competing interests.

## Authors' contributions

EN developed the model, performed software implementation and drafted the manuscript. EB conceived of the study and participated in coordination. All authors read and approved the final manuscript.

## Supplementary Material

Additional file 1 **Kernel functions**. Rationale for using the kernel functions from Table 1. Click here for file

Additional file 2 **Identifiability note**. Discussion on the parameter identifiability for the developed models. Click here for file

Additional file 3 **Modified forward selection (FS) algorithm**. Description and testing of the modified version of the FS algorithm. Click here for file

Additional file 4 **Adaptive model selection (AMS)**. Description of the AMS algorithm to identify the kernel function that reconstructs the prior links with the highest accuracy. Click here for file

Additional file 5 **Simulated and experimental data**. Details on the artificial and real systems used for testing and description of the testing procedure. Click here for file

Additional file 6 **Independent artificial data**. Testing of the AMS algorithm using independent set of artificial data described in [5]. Click here for file

Additional file 7 **Discussion**. Discussion and perspectives for further research. Click here for file
