# Supplementary material for: Model selection in the reconstruction of regulatory networks from time-series data
Source: BMC Res Notes. 2009 May 5;2:68. doi: 10.1186/1756-0500-2-68 (PMC2688516; doi:10.1186/1756-0500-2-68)
Supplement: Additional file 1 — Kernel functions. Rationale for using the kernel functions from Table 1. [file 1756-0500-2-68-S1.pdf]

Additional file 1: Kernel functions

In this paper, we are not aiming at imposing specific state-space models for the network inference. Rather, the presented, in Table 1, models should be considered as a set of reasonable approximations that can cover broad range of processes.

The polynomial models (P1 and P2) originate from the Taylor expansion of the kernel function, if we keep just one (P1) or two (P2) terms in the expansion. Note that integrating the linear ordinary differential equations model (5) also yields the integral model with the zero-degree polynomial kernel (P1) [1]. The rational functions (I1, I2 and I3) are based on the Padé approximants that are derived by expanding a function as a ratio of two power series [2]. Even though the exponential models (E1, E2 and E3) can also be considered as approximations for a broad range of decaying behaviours, they correspond to well-defined state-space models too. Single exponential models (E1 and E2) represent solutions for the simplest, one-state, state-space model (one square in the control nodes in Fig 1). Bi-exponential model (E3) may correspond to the state-space model with two states placed in parallel (as in control node 1 in Fig 1) or in a chain (as in control node 2 in Fig 1).

In applications, we prefer to keep τ_l fixed in models (7) and (8) as it leads to the models linear with respect to the unknown parameters ($u_{l,ij}$). These models can be represented as linear regression models allowing us to directly compute the best-fit parameters from the data. It is also straightforward to apply non-linear models, but these models lead to non-linear regression requiring computationally intensive, iterative approaches. Therefore, we generally prefer to use linear models unless we have strong evidence or prior knowledge that a model should be non-linear. The characteristic decay times, τ_l, in models (7) and (8) are selected to approximate relatively rapid ($0.1T$) and relatively slow ($0.9T$) processes occurring in a system, where T is the last time point in a time series.

This library is by no means complete and may be extended and optimized in a number of ways. In particular, new models can be created once more information is available on molecular interactions. It is also possible to develop new approximations for $w_{ji}(t)$ for empirical validation on the systems with known outcome.

References

1. Novikov E, Barillot E: **Regulatory network reconstruction using an integral additive model with flexible kernel functions.** *BMC Systems Biology* 2008, **2**: 8.
2. Baker GA, Graves-Morris P: **Padé Approximants**. Cambridge University Press. 1996.
