# Supplementary material for: Model selection in the reconstruction of regulatory networks from time-series data
Source: BMC Res Notes. 2009 May 5;2:68. doi: 10.1186/1756-0500-2-68 (PMC2688516; doi:10.1186/1756-0500-2-68)
Supplement: Additional file 2 — Identifiability note. Discussion on the parameter identifiability for the developed models. [file 1756-0500-2-68-S2.pdf]

Additional file 2: Identifiability note

Once the model is established, one should investigate if the underlying parameters defining the model (and therefore the network structure) can be determined unambiguously from experimental data. This is the subject of the identifiability analysis. In general, this may be a difficult problem, deserving a separate publication. In our case, however, the identifiability analysis is rather straightforward. The forward selection (FS) procedure starts with no links in the system and it consequently adds new links trying to approximate behaviour of the nodes with the lowest fitness. Taking into account that the inference regulatory model (4) is additive and the kernel functions are defined by (6), (7) and (8), it is easy to see that the model parameters used to approximate behaviour of each node are uniquely identifiable. In practical applications, however, we can consider two cases, when the models may become unidentifiable. For short time series, the number of unknown model parameters may exceed the number of experimental time points. In this case, addition of a new link does not improve the node fitness. The FS procedure can report on that and then it may either stop or continue to approximate other nodes. Alternative approach would be to limit number of links that can be associated with each node. Another case of practical unidentifiability is when different nodes demonstrate similar dynamics such that FS procedure can not distinguish between similar nodes and therefore it may select a wrong node as a regulator. This problem is particularly important for short time series and small signal-to-noise ratios. As shown in the paper, this may be the main reason why the performance of the network reconstruction was relatively poor for the yeast glycolysis pathway and for the experimental *cdc15* dataset. In this case, similar nodes can be collected in a cluster (using one of the clustering techniques) and the network should be defined between the clusters. In this paper, we do not explore further this clustering step considering it as a separate topic of research.

In principle, model identification can be done via direct parametric fit of some generic model to experimental data. However, in this case, the amount of unknown parameters may be as large as the number of measured data points, leading to huge uncertainties about the reconstructed networks. Simpler models approximate main trends in system behaviour filtering out weaker distortions. More complicated models can be distracted by these distortions giving arbitrary results. Therefore, we prefer to develop a number of simple models, which can approximate system responses reasonably well. Each model is characterized by a relatively small number of parameters that can be easily estimated from the data. These parameters are further used to reproduce the network structure.
