# Supplementary material for: Model selection in the reconstruction of regulatory networks from time-series data
Source: BMC Res Notes. 2009 May 5;2:68. doi: 10.1186/1756-0500-2-68 (PMC2688516; doi:10.1186/1756-0500-2-68)

Additional file 3: Modified forward selection (FS) algorithm

An additional confounding factor that may influence the network reconstruction is the fitting algorithm itself. It is well known that the FS procedure can easily be trapped in the local minima. If a wrong node is selected at an early iteration because it gives the best quality of fit for the selected node, the decision cannot be reconsidered at later iterations taking into account additional links created after that wrong decision. Therefore, we have modified the original FS procedure by testing if the removal of links would improve the node fitness. More specifically, we test two possibilities at any iteration. One is, as before, addition of a single link. The other one is removal of one link and addition of two other links instead. The modification that leads to the highest fitness for the selected node remains in the system. We applied the modified FS algorithm for the network reconstruction of the three artificial systems [Additional file 5]. The dependencies of positive predictive value (PPV) on the total number of links are presented in Fig. S1. One can see that, although the modified version of the FS algorithm led to somewhat better performance in all cases, the main difference originated from the difference in the models. As the modified FS algorithm was notably slower than the original one and the gain in performance is not that high, we will use further the original FS procedure. Nevertheless, we stress that search for more appropriate fitting algorithms should complement model development.

Figure S1 - The average dependencies of PPV on the total number of links for the three artificial systems using the original and modified versions of the FS algorithm.
Solid lines correspond to the original version and dashed lines correspond to the modified version of the FS algorithm. Blue line is for the linear ordinary differential equations model (5), red line is for the E3 model and green line is for the I3 model. Dashed black line corresponds to random prediction. Confidence intervals for the obtained estimates are too narrow to be recognizable in the graphs and therefore not shown.

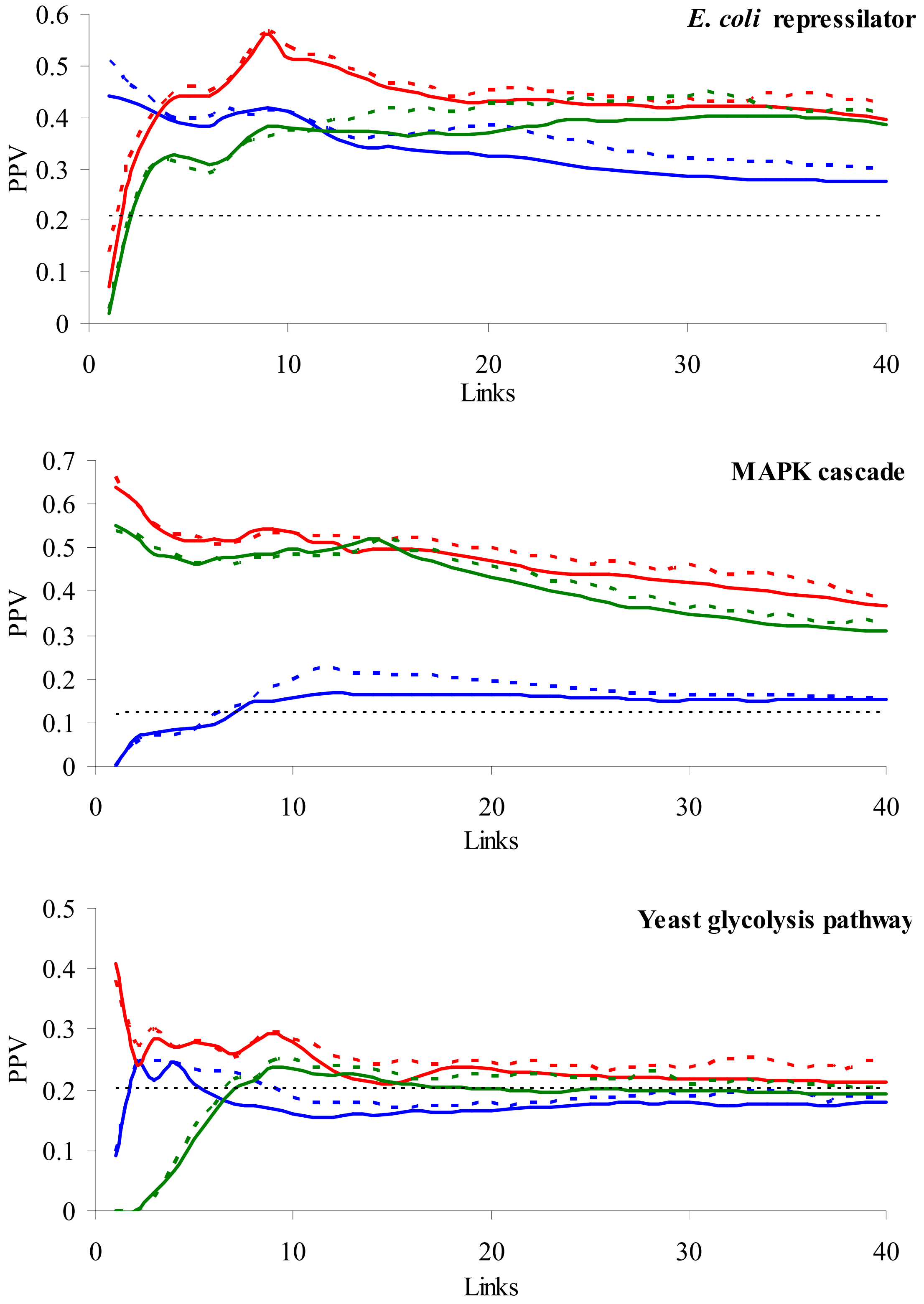

Supplement: Additional file 3 — Modified forward selection (FS) algorithm. Description and testing of the modified version of the FS algorithm. [file 1756-0500-2-68-S3.pdf]
