# Supplementary material for: Model selection in the reconstruction of regulatory networks from time-series data
Source: BMC Res Notes. 2009 May 5;2:68. doi: 10.1186/1756-0500-2-68 (PMC2688516; doi:10.1186/1756-0500-2-68)
Supplement: Additional file 4 — Adaptive model selection (AMS). Description of the AMS algorithm to identify the kernel function that reconstructs the prior links with the highest accuracy. [file 1756-0500-2-68-S4.pdf]

Additional file 4: Adaptive model selection (AMS)

We can use prior knowledge on the nodes' interactions to select the best network reconstruction model from the pre-defined library. For the current study, the model library is presented in Table 1. We look for the kernel function $w_{ij}(t)$ that reconstructs the prior links with the highest accuracy. The algorithm is as follows:

1. Define the models to be tested (Table 1);
2. Select the number of iterations (NI) of the forward selection (FS) procedure. We set NI equal to the number of prior links as we hope that these prior links should be recovered at the earlier FS iterations.
3. After NI iterations, we count the number of correct links recovered by each of the models from Table 1. The model that found the maximal number of correct links is selected for further reconstruction, and the search is stopped.
4. If neither of the models is able to find any correct link, we increase NI and perform FS again. We continue increasing NI until a user-defined limit for NI is achieved.
5. If it happens that two (or more) models find the same, maximal, number of correct links, we increase NI and run FS only for the best models. NI is increased until one of the models wins or until a user-defined limit for NI is bypassed.
6. If neither of the models wins, even after the user-defined NI limit, the "best" model is selected at random.
7. The selected model is then applied for the final reconstruction.

The complexity of the algorithm scales linearly with the number of nodes. This is defined by the FS algorithm. At each of the FS iterations, the algorithm should find two nodes to create a link: one node that has the lowest fitness (largest deviation from the observed behaviour) and another node that can improve the fitness of the first node better than the others. The number of FS trials is therefore proportional to the number of nodes in a system.
