# Supplementary material for: Model selection in the reconstruction of regulatory networks from time-series data
Source: BMC Res Notes. 2009 May 5;2:68. doi: 10.1186/1756-0500-2-68 (PMC2688516; doi:10.1186/1756-0500-2-68)
Supplement: Additional file 5 — Simulated and experimental data. Details on the artificial and real systems used for testing and description of the testing procedure. [file 1756-0500-2-68-S5.pdf]

Additional file 5: Simulated and experimental data

We compared the performances of the eight kernel functions from Table 1 as well as the linear ordinary differential equations regulatory model (5) using simulated and experimental data. The integral equations model with the zero-degree polynomial (P1) and the single exponential (E2) kernels has been also thoroughly tested and compared to the dynamic Bayesian networks approach in [1].

Three artificial systems defined by mathematical models for real biological networks were used for testing: the oscillating network in *E. coli*, called repressilator [2], the mitogen-activated protein kinase (MAPK) cascade [3] and the glycolysis pathway in yeast [4]. These models can be imported in JDesigner [5] as SBML modules [6, 7] and used to generate idealistic time series. We stress that we used these modules as they were originally developed *i.e.* without any modifications in the structure or in the kinetic parameters of the models. We used JDesigner to integrate the models on 100 time points spaced uniformly over the interval [0;1000] for repressilator, [0;1] for yeast glycolysis and [0;100] for the MAPK cascade. Two data distorting steps were performed: we sampled the generated time series leaving 20 time points at random time intervals and added Gaussian noise with the noise-to-signal level equal to 0.05. The SBML files and examples of time series can be found on our web page [8].

We used the yeast (*Saccharomyces cerevisiae*) cell cycle microarray time-series data [9] to demonstrate applicability of the developed approach to real experimental data. This dataset consists of three subsets measured using different cells synchronization methods: α factor-based (*alpha*, 18 time points), size-based (*elu*, 14 time points) and cdc15-based (*cdc15*, 24 time points). The yeast cell cycle pathway is available from KEGG [10]. Different researchers [11 ,12, 13] tested their algorithms reconstructing sub-networks of this pathway. We followed this way and selected a 16-gene sub-network shown on our web page [8]. Then we could

evaluate the performance of the algorithms by comparing reconstructed networks with the known network in the same way as it is possible for artificial systems.

When the true networks' structures are known, we can count the number of true positive (TP), true negative (TN), false positive (FP) and false negative (FN) links. Different performance parameters can be further derived [14, 15] from these four numbers. As in [5], we use the positive predictive value (PPV) and sensitivity (Se) defined as PPV = TP/(TP+FP) and Se = TP/(TP+FN). Each time the FS procedure adds a link we update the number of TP, FP and FN and calculate PPV and Se. This creates the dependence of the performance (PPV and Se) of the inference models on the number of generated links. The resulting values of PPV and Se are averaged over 100 runs of the simulation procedure. For artificial systems, the simulation procedure generates different time sampling and different realizations of noise at each run whereas the network structure, kinetic laws and kinetic parameters remain the same. We also randomly generate prior links used in testing of the model selection algorithm. This is certainly not the most realistic way of link selection. Typically, a few really important links that can be detected using other techniques are known. Nevertheless, we prefer to select prior links at random to avoid any subjective bias. The real system has no parameters to be randomly generated. Therefore, we performed 100 runs only for testing the model selection algorithm where, at each run, only prior links are randomly generated.

The developed software for network inference is freely available on our web page [8]. Installation also contains a detailed step-by-step user manual.

References

1. Novikov E, Barillot E: **Regulatory network reconstruction using an integral additive model with flexible kernel functions.** *BMC Systems Biology* 2008, **2**: 8.

2. Elowitz MB, Leibler S: **A synthetic oscillatory network of transcriptional regulators.** *Nature* 2000, **403**: 335-338.

3. Huang CHF, Ferrell Jr JE: **Ultrasensitivity in the mitogen-activated protein kinase cascade.** *Proc. Natl. Acad. Sci. USA* 1996, **93**: 10078-10083.

4. Pritchard L, Kell DB: **Schemes of flux control in a model of Saccharomyces cerevisiae glycolysis.** *Eur. J. Biochem.* 2002, **269**: 3894-3904.

5. **JDesigner** [http://sbw.kgi.edu/software/jdesigner.htm]

6. **Cellular Models: Model Repository** [http://www.cds.caltech.edu/~hsauro/models.htm]

7. **BioModels Database** [http://www.ebi.ac.uk/compneur-srv/biomodels-main/publ-models.do]

8. **NETI (Network Inference) Download** [http://bioinfo.curie.fr/projects/reverse-engineering/]

9. Spellman PT, Sherlock G, Zhang MQ, Iyer VR, Anders K, Eisen MB, Brown PO, Botstein D, Futcher B: **Comprehensive identification of cell cycle-regulated genes of the yeast Saccharomyces cerevisiae by microarray hybridization.** *Mol Biol Cell* 1998, **9**:3273-3297.

10. **KEGG Cell cycle-yeast-Saccharomyces cerevisiae** [http://www.genome.jp/kegg/pathway/sce/sce04111.html].

11. Wu CC, Huang HC, Juan HF, Chen ST: **GeneNetwork: an interactive tool for reconstruction of genetic networks using microarray data.** *Bioinformatics* 2004, **18**: 3691-3693.

12. Kim SY, Imoto S, Miyano S: **Dynamic Bayesian network and nonparametric regression model for inferring gene networks.** *Genome Informatics* 2002, **13**: 371-372.
13. Sokhansanj BA, Fitch JP, Quong JN, Quong AA: **Linear fuzzy gene network models obtained from microarray data by exhaustive search.** *BMC Bioinformatics* 2004, **5**:108.
14. Soranzo N, Bianconi G, Altafini C: **Comparing association network algorithms for reverse engineering of large-scale gene regulatory networks: synthetic versus real data.** *Bioinformatics* 2007, **23**: 1640-1647.
15. Bansal M, Belcastro V, Ambesi-Impiombato A, di Bernardo D: **How to infer gene networks from expression profiles.** *Molecular Systems Biology* 2007, **3**: 78.
