# Supplementary material for: Model selection in the reconstruction of regulatory networks from time-series data
Source: BMC Res Notes. 2009 May 5;2:68. doi: 10.1186/1756-0500-2-68 (PMC2688516; doi:10.1186/1756-0500-2-68)
Supplement: Additional file 6 — Independent artificial data. Testing of the AMS algorithm using independent set of artificial data described in [5]. [file 1756-0500-2-68-S6.pdf]

Additional file 6: Independent artificial data

We explored the performance of the adaptive model selection (AMS) algorithm using an independent set of artificial data described in [1]. Briefly, 20 random 10-gene networks with an average in-degree per gene of 2 were generated. For each network, time-series data (1000 time points) were simulated using linear ordinary differential equations. Each data point was statistically distorted with noise-to-signal ratio equal to 0.1. In our analysis, we extracted the last 20 time points from the 1000-point time series, which were then used for network reconstruction. For model selection, we used models from table 1 that needed estimation of only one parameter (P1, E1, E2, I1 and I2), and we skipped bi-parametric models (P2, E3 and I3). As the network structures were known, we used, as before, 2 and 10 prior links for model identification. We counted number of times each model was selected in the 100 runs of the network reconstructions. At each run, the prior links were randomly generated. The obtained results were further averaged over the 20 available networks. The results of model selection using 2 and 10 prior links are presented in Fig. S2.

As the first-order linear ordinary differential equations were used in simulations, we presume that the P1 model should be the most appropriate model for reconstruction. Indeed, with 10 prior links, the P1 model was an unambiguous winner. With 2 links, its performance was expectantly weaker. However, even in this case, only the E1 model could compete with the P1 model, leading to more precise model identification. Besides limited number of randomly selected prior links, wrong selection can be also due to the statistical noise distorting data or due to the specifics of the numerical implementation of the underlying dynamical model.

Figure S2 - Adaptive model selection links for the independent set of artificial data [1].
Number of times each single-parametric model from Table 1 (P1, E1, E2, I1 and I2) has been selected in the 100 runs of the model reconstruction by the AMS algorithm based on 2 (empty bars) and 10 (filled bars) prior links. At each run, the prior links were randomly generated. Confidence intervals for the random model selection are indicated by dashed lines.

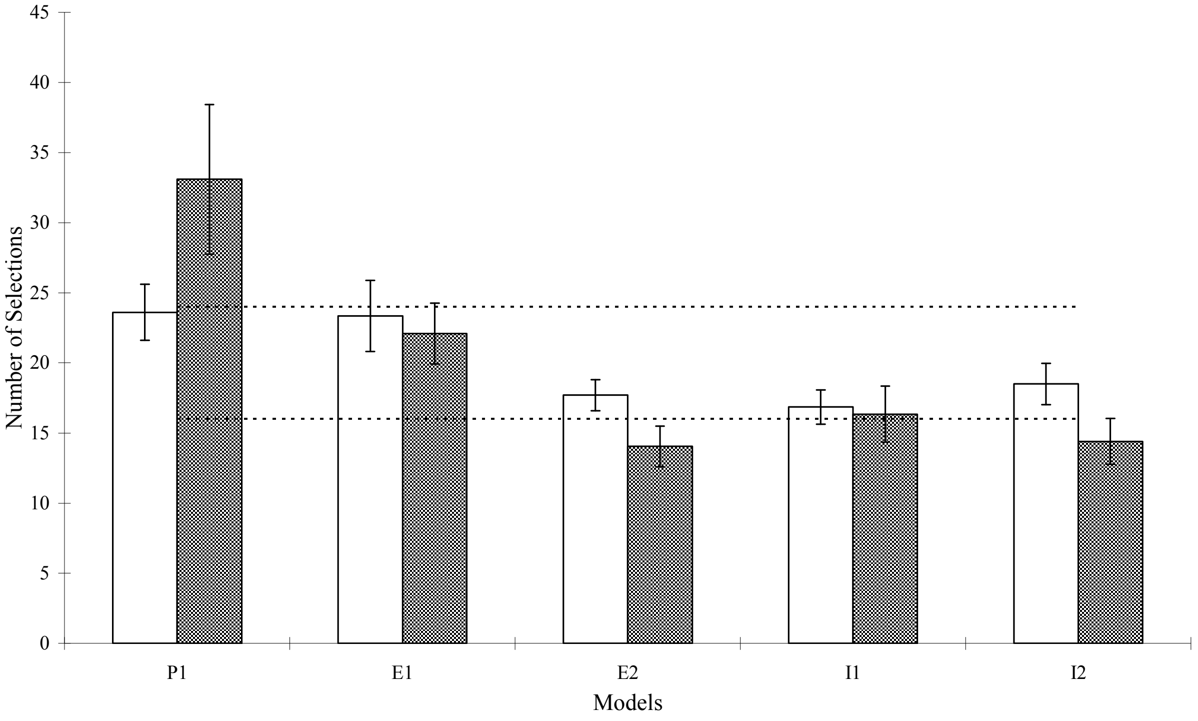

References

1. Bansal M, Belcastro V, Ambesi-Impiombato A, di Bernardo D: **How to infer gene networks from expression profiles.** *Molecular Systems Biology* 2007, **3**: 78.
