# Supplementary material for: Model selection in the reconstruction of regulatory networks from time-series data
Source: BMC Res Notes. 2009 May 5;2:68. doi: 10.1186/1756-0500-2-68 (PMC2688516; doi:10.1186/1756-0500-2-68)
Supplement: Additional file 7 — Discussion. Discussion and perspectives for further research. [file 1756-0500-2-68-S7.pdf]

Additional file 7: Discussion

We have presented a generalized approach for the regulatory network reconstruction where integral equations are used to model interactions between the observable nodes (genes, proteins, etc.) of a network. Each integral is defined by a kernel function originated from the state-space formalism. The advantage of the developed approach is its flexibility as we can switch between different models just by changing the kernel function or maybe even by changing a few parameters characterizing the kernel. This gives us an easy possibility to create and to test different inference models and, potentially, to identify appropriate models from experimental data. In principle, model identification can be done via direct parametric fit of some generic model to experimental data. However, in this case, the amount of unknown parameters may be as large as the number of measured data points, leading to huge uncertainties about the reconstructed networks. Simpler models approximate main trends in system behaviour filtering out weaker distortions. More complicated models can be distracted by these distortions giving arbitrary results. Therefore, we prefer to develop a number of simple models, which can approximate system responses reasonably well. Each model is characterized by a relatively small number of parameters that can be easily estimated from the data. These parameters are further used to reproduce the network structure. Model identification in this case can be performed based on how well each of the developed models can reconstruct the known links. We have shown that even with as small as two prior links it is already possible to select models ensuring reasonable performance. This approach can be considered as a regularization technique allowing for more robust, although somewhat biased (leading to false positives), analysis.

An alternative approach would be to apply consequently different models and to look for the links that are reconstructed by the most of the models (majority voting). In our experiments, however, this approach could not improve the performance. This conclusion has been also

recently supported in [1] where several other network reconstruction algorithms were tested and compared. Another attractive possibility would be to have a unique kernel function that could approximate broad range of systems. However, as we have shown, using the same model for any system can lead to very poor predictions. Therefore, we are obliged to look for specific models that are best adopted for each particular system.

In this paper, we explored networks with ~20 nodes. Although it is straightforward to apply the developed approach for larger networks, we can hardly expect to achieve high performance using short (~20 points) time series. As the number of observable nodes increases, it becomes more difficult for the inference algorithm to distinguish between time series corresponding to different nodes. In addition, the problem of network inference becomes heavily underdetermined: the number of unknown parameters largely exceeds the number of experimental points. To reduce the uncertainty, the number of observable nodes should be decreased either by selecting specific sub-networks or using clustering approaches.

Network inference depends on the "distance" between the inference and data-generating models. Even if the same model is used for any link in data generation, this model remains unknown. If all inference models are different from the true one, the obtained performance can be similar, and probably non-optimal, for these models (like, for example, for the MAPK cascade). In this case, however, the "correct" model can be found by extending the library of inference models (Table 1). If, in data generation, different models are used for each link, any unique inference model may be almost equivalently far from any link model resulting, again, in the performance independent of the inference model. Looking for a unique, link-independent, inference model could hardly be successful in this case. New approaches should allow for more flexible identification of the link models within the same system. This requires the testing of a huge number of model combinations with the computational costs dependent on the size of experimental datasets, number of nodes, model library and implementation of the fitting algorithm. This approach needs more careful elaboration than by using brute force,

combinatorial, approaches. We would also need more experimental data to be able to identify specific link models directly from the data. Prior knowledge would allow us to formalize certain nodes' interactions and thus to decrease the search space.

Another important perspective is to accompany model developments by the development of fitting algorithms. The forward selection (FS) is fast and easy to implement, but it is obvious that more robust algorithms that can avoid local minima are needed to improve the overall performance of the network reconstruction. One modification of the original FS algorithm has been discussed in this paper [Additional file 3]. Although it could slightly improve the performance, it is still far from the ideal and further efforts are clearly necessary in this direction.

Finally, we note that networks derived from limited experimental data should only be considered as rough approximations for real network structures. New experiments should be designed to yield datasets to improve the reconstruction. The initially derived network can be used to optimally design these new experiments. This should lead to the improved identification of the network structure with less experimental effort and expenses. This, however, remains a subject of further research.

References

1. Bansal M, Belcastro V, Ambesi-Impiombato A, di Bernardo D: **How to infer gene networks from expression profiles.** *Molecular Systems Biology* 2007, **3**: 78.
